# The Effect of Recycled Spent Coffee Grounds Fertilizer, Vermicompost, and Chemical Fertilizers on the Growth and Soil Quality of Red Radish (*Raphanus sativus*) in the United Arab Emirates: A Sustainability Perspective

**DOI:** 10.3390/foods13131997

**Published:** 2024-06-25

**Authors:** Athari K. Mesmar, Shaikha T. Albedwawi, Aysha K. Alsalami, Alreem R. Alshemeili, Abdelghafar M. Abu-Elsaoud, Khaled A. El-Tarabily, Seham M. Al Raish

**Affiliations:** 1 Department of Biology, College of Science, United Arab Emirates University, Al Ain 15551, United Arab Emirates; 201901356@uaeu.ac.ae (A.K.M.); 201900644@uaeu.ac.ae (S.T.A.); 201907925@uaeu.ac.ae (A.K.A.); 201800823@uaeu.ac.ae (A.R.A.); ktarabily@uaeu.ac.ae (K.A.E.-T.); 2 Department of Botany and Microbiology, Faculty of Science, Suez Canal University, Ismailia 41522, Egypt; abuelsaoud@science.suez.edu.eg; 3 Department of Biology, College of Science, Imam Mohammad Ibn Saud Islamic University (IMSIU), Riyadh 11623, Saudi Arabia

**Keywords:** fertilizer impact analysis, food wastes, organic farming, plant growth promotion, soil conditions, soil fertility, sustainable agriculture

## Abstract

The overuse of chemical fertilizers degrades the soil ecosystem and restricts the natural development of plants. Various byproducts are produced throughout the production and consumption of coffee within the coffee industry, and they are significant in terms of environmental waste. Spent coffee grounds (SCGs) contains various bioactive compounds that have demonstrated potential applications in various fields. These compounds can enhance soil quality by improving its physicochemical properties and biological fertility, ultimately leading to improved plant growth and reducing food waste and contamination at the same time. This current study examined the impact of chemical fertilizer, vermicompost, SCGs with percentage fertilizer (SCGPF), and SCGs on the top dressing fertilizer (SCGTDF) on red radish (*Raphanus sativus*) growth and soil quality. This greenhouse experiment tested various concentrations of SCGPF (5%, 10%, 25%, and 50%) and different doses of SCGTDF (0.5 g, 1 g, and 2.5 g). The results showed that the 0.5 g SCGTDF treatment yielded the highest mean plant length (18.47 cm) and fresh weight (27.54 g), while the vermicompost at a 50% concentration produced the highest mean leaf surface area (58.32 cm^2^). These findings suggest the potential of SCGs as a sustainable fertilizer alternative, contributing to improved plant growth and soil quality, thus supporting sustainable agricultural practices and a circular economy.

## 1. Introduction

Spent coffee grounds (SCGs) are food waste produced in large quantities, with an annual generation of 15 million tons. They contain substances such as tannins, caffeine, and phenols [1]. Adding SCGs to the soil enhances its chemical and physical properties, increasing the levels of nitrogen (N), phosphorus (P), and potassium (K). It also enhances the structural stability of the soil aggregates, water-holding capacity, and soil hydrophobicity, improving the soil organic matter content, decreasing the soil bulk density, and increasing the soil microbial diversity [1,2,3,4]. This feature makes SCGs an effective solution for various soil types that frequently exhibit a significant deficiency of organic matter, rendering them highly vulnerable to erosion, such as in the United Arab Emirates (UAE) [5,6,7]. Although a significant amount of organic waste from SCGs undergoes recycling, the disposal rates remain high. Hence, pursuing strategies to reduce these rates is considered immensely valuable. The utilization of SCGs has experienced a significant increase in popularity in recent years, primarily because they contain a high concentration of organic compounds such as fatty acids, lignin, cellulose, hemicellulose, and other polysaccharides [8]. Studies have explored their potential application in improving soil fertility and increasing agricultural productivity [8].

Simultaneously, the worldwide urban population is projected to reach 8.6 billion by 2030, leading to a rise in food and waste production demands [9]. This creates a sense of urgency and becomes a crucial obstacle for sustainable management, necessitating inventive solutions that address environmental issues and enhance crop production quality and quantity [9,10]. This issue can be resolved by implementing a promising and encouraging strategy that focuses on repurposing different food waste materials, including SCGs, to produce organic fertilizers with fewer chemical side effects [1,2,4,6,11]. Several studies have explored this approach [11,12,13,14,15,16], demonstrating its potential to mitigate environmental impacts while improving soil health and crop yields [12,14,16].

Vermicompost (VC) is an example of using food waste as fertilizers. It is manufactured through the interaction between earthworms and microorganisms, accelerating the breakdown of organic matter [17,18]. This process, known as biodegradation, rapidly converts unstable organic matter into stable humus through complete oxidation. The VC derived from biowaste can be repurposed as fertilizers or substrates with a high nutrient content [17]. These materials are finely fragmented, resemble peat, and possess a high porosity, aeration, drainage, and water-holding capacity [17,18]. Research has demonstrated that incorporating VC into soils can enhance the germination, growth, and yields of diverse vegetables, ornamentals, and other crops, including cowpeas, cress, grapes, bananas, strawberries, and tomatoes [18,19,20].

In contrast, chemical fertilizers pose a significant environmental concern in agricultural production [7,14,20]. Global food production heavily relies on synthetic chemical fertilizers [14,21]. Recently, the world has recognized the importance of sustainable agriculture, which is defined as production that is based on minimal resource expenses and pollution to maintain environmental resilience [21,22]. Chemical fertilizers are used to improve the plant growth and to provide nutrients. In contrast to organic fertilizers, the overuse of chemical fertilizers can lead to soil degradation, water pollution, and greenhouse gas emissions [21,22]. It is important to use fertilizers responsibly to maximize their benefits while minimizing their negative environmental impacts [7,13,17,22,23,24,25].

Organic red radish (*Raphanus sativus*) is an annual root vegetable crop from the family Brassicaceae. The roots, leaves, and sprouted seeds of the red radish may be ingested raw or in salad [24]. The radish root epidermis has various colors (white, red, pink, purple, and yellow), but the root flesh is white and pungent, with a crisp flavor [24]. Due to the anthocyanin pigment, the root skin is crimson. A sufficient amount of vitamins, glucosinolates, sulforaphane, polyphenolic compounds, sulfur, calcium, potassium, and phosphorus are present in the root, and it is associated with many significant health benefits [24] due to the presence of biologically active and potent anti-oxidant substances [25]. Melchini and Traka [26] found that these substances may aid in reducing or preventing the risk of various diseases, including cardiovascular disease, certain malignancies, hypertension, strokes, and other chronic diseases [21,25,26].

This current study introduces a novel approach to reduce the dependency on synthetic agrochemicals by exploring using SCGs as fertilizer. Conducted in controlled greenhouse conditions, this research assessed the effects of SCGs on the biomass and soil quality of radish plants. This marks the first such investigation in the UAE, targeting both the growth measurements of the biomass of radish plants and the soil health. This study could also promote a circular economy by reusing agri-food byproducts. This research supports reducing synthetic fertilizer use and mitigates the environmental impacts of their production and use.

## 2. Materials and Methods

### 2.1. Experimental Site and Design

This current study was conducted in the greenhouse of the United Arab Emirates University, College of Science, Biology Department (GPS 24.200250579134575, 55.67575985048151), during the winter seasons (January to April) of 2023.

The experiment was set in a split–split–plot design, where SCGs, VC, chemical fertilizer (CF), and the control were randomly distributed within the sub-plots. Each experimental sub-plot consisted of eight rows, with three replicates in random order. In each pot, there were two seeds.

The fertilizers were evaluated on organic red radish seedlings (*R. sativus*). We aimed to test the efficacy of the SCGs compost fertilizer, VC, and CF to control the growth rate and biomass measurement of the radish and the soil quality. In these experiments, the fertilizers and control used were prepared as follows.

#### 2.1.1. Control (C)

A seed starter potting mix, manufactured by Gardener’s in the United Arab Emirates, was used as the control treatment with the following product specifications: the basic material (dry soil) was decomposed plant material; the density was >200 kg/m^3^; the organic matter was 88%; the moisture content was 47%; the electrical conductivity (EC) was <1.5 millimhos per centimeter (mmhos/cm); the salt content was <1.5 g/L, and the pH was 5.5–6.5. The optimal EC range for growing radish is 1.0–1.5 ms/cm (1000–1500 μs/cm) [27].

#### 2.1.2. SCGs

SCGs gathered from the canteen of the United Arab Emirates University were exploited in two distinct ways. In the first approach, SCGs percentage fertilizer (SCGPF) was used. This involved mixing specific proportions of SCGs with the seed starter potting mix from the first day: (1) SCGPF 5 = 5% (5 parts of SCGs and 95 parts of seed starter potting mix), (2) SCGPF 10 = 10% (10 parts of SCGs and 90 parts of seed starter potting mix), (3) SCGPF 25 = 25% (25 parts of SCGs and 75 parts of seed starter potting mix), and (4) SCGPF 50 = 50% (50 parts of SCGs and 50 parts of seed starter potting mix).

The second method, known as SCGs on the top dressing fertilizer (SCGTDF), involved applying SCGs to the top layer of the soil. This method was implemented during the second and third weeks after planting the seeds, using three different quantities of SCGTDF: (1) SCGTDF 0.5 g = 0.5 g of SCGs on the top surface layer of the soil, before each pot was irrigated with 50 mL of water; (2) SCGTDF 1 g = 1 g of SCGs on the top surface layer of the soil, before each pot was irrigated with 50 mL of water; and (3) SCGTDF 2.5 g = 2.5 g of SCGs on the top surface layer of the soil, before each pot was irrigated with 50 mL of water. The total volume for the pots were 1 kg of seed starter potting mix and treatments.

The physicochemical characteristics of the SCGs were as follows: a pH of 5.5, an EC of 9.05 dS/m, 50.5% of carbon, and 2.4% of nitrogen. The SCGs also consisted of the following elements in mg/kg: phosphorus, 1450; potassium, 9600; Mg, 1950; S, 1550; Ca, 1250; Fe, 54; Mn, 30; Cu, 20; Co, 16; and Zn, 9.

#### 2.1.3. VC

Vermicomposting is a natural process whereby earthworms (*Eisenia fetida*) known as red wigglers convert waste material with rigid structures into compost. Mixed food waste was used to feed earthworms. Then, the VC products were mixed with the seed starter potting mix in different percentages from the first day as follows: (1) 10% V = 10 parts of VC product and 90 parts of seed starter potting mix; (2) 25% V = 25 parts of VC product and 75 parts of seed starter potting mix; and (3) 50% V = 50 parts of VC product and 50 parts of seed starter potting mix.

#### 2.1.4. CF

In total, 1 g of chemical fertilizer was mixed with 1000 mL of water to create a 1.2 EC solution. It was applied in the second and third weeks after planting the seeds. The product was a powder composed of 20% of N, 20% of P_2_O_5_, and 20% of K_2_O + microelements. It was applied by fertigation [28].

### 2.2. Greenhouse Experiments

In the greenhouse experiments, all the above treatments were used. For each treatment/group, eight separate pots, each containing two seeds, were arranged in a split–split design. The greenhouse experiments were repeated three times. The control and inoculated soil were maintained in the greenhouse (15 h day/9 h night) for 35 days at a temperature of 28 ± 2 °C and a relative humidity of 42 ± 5%. Table 1 shows the treatments employed in this current study, including the control group, CF, VC, SCGTDF, and SCGPF.

### 2.3. Plant Growth Measurements

The following plant parameters were measured:

#### 2.3.1. Radish Height

1. The height of the whole fresh radish (including the shoot, leaves, and roots) using a tape measure with cm as the unit.
2. The height of the shoot of the fresh radish by using a tape measure with cm as the unit.
3. The height of the root of the fresh radish by using a tape measure with cm as the unit.
4. The taproot top perimeter of the fresh radish root using a tape measure with cm as the unit.

#### 2.3.2. Radish Weight

Using an analytical weighing scale, the total weight of the fresh and dried radish was determined.

#### 2.3.3. Leaf Surface Area (LSA)

The grid count approach, which takes the dimensions of the leaf (the width and length) into account, was used to measure LSA. Cm^2^ was the unit of measurement [29].

### 2.4. Effect of Different Treatments on the Soil’s pH and EC

The soil quality was determined by measuring the pH and EC of the soil. The pH and EC were measured using an HEM conductivity meter from Technical Jica (Japan Cooperation, Tokyo, Japan) [30,31].

### 2.5. Determination of the Soil’s Macronutrients

The chemical characteristics of the soil were analyzed and are listed in Table 2. The organic carbon (C), available P and K, and N were measured as described by [32].

### 2.6. Determination of the Total Bacterial Population in the Soil Samples

The soil dilution plate method was used to enumerate the total bacteria from each soil sample using a nutrient agar medium (NA) (Lab M Limited, Lancashire, United Kingdom) [33]. The NA medium was supplemented with nystatin and cycloheximide (50 µg mL^−1^ of each; Sigma-Aldrich Chemie GmbH, Taufkirchen, Germany). These two antifungal antibiotics were mixed with the NA just before pouring the plates. Ten grams of each soil and 100 mL of sterile deionized water were mixed in Erlenmeyer flasks. The soil suspension was then agitated at 28 °C for 30 min using a rotary shaker (Model G76, New Brunswick Scientific, Edison, NJ, USA) set to 250 revolutions per minute (rpm). The flasks were shaken, and 0.2 mL portions were then diluted tenfold (10^−2^–10^−5^) with sterile deionized water and spread over NA plates using a sterile glass spreader [33]. The total bacterial population in every soil sample was calculated [33].

### 2.7. Statistical Analyses

The data normality was checked using Shapiro–Wilk normality testing at a level of 0.05. The difference between the treatment groups was performed using a one-way analysis of variance (ANOVA). The overall difference, influenced by different factors, was assessed by a multivariate ANOVA (MANOVA). The ANOVA was followed by the Duncan’s Multiple Range Test. All the statistical analyses were performed at 0.05, 0.01, and 0.001 significance levels. The statistical package for social sciences (SPSS 29; SAS Institute Inc., Cary, NC, USA) was used for the data analysis. 

## 3. Results

### 3.1. Effect of Different Treatments on R. sativus Growth Characteristics

**Figure 1:**
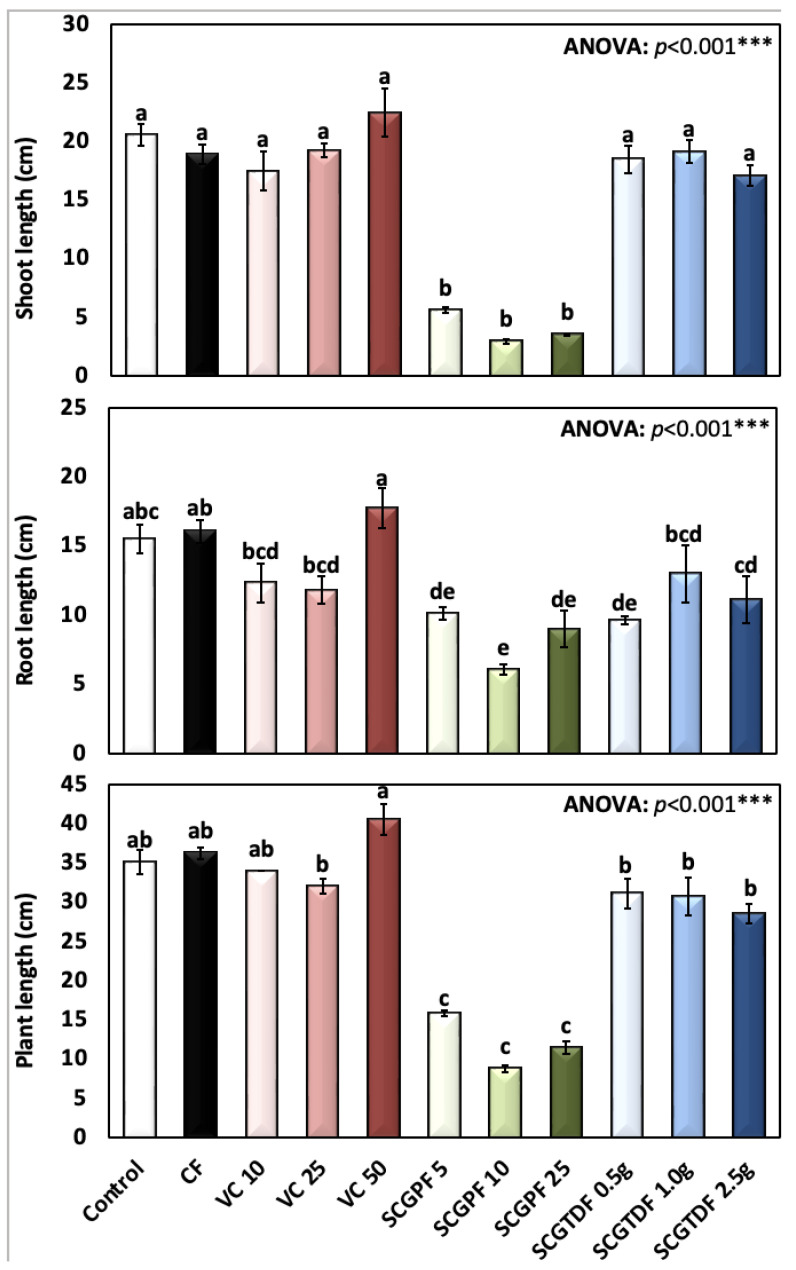
Shoot length (cm), root length (cm), and total plant length (cm) of *R. sativus* treated with different treatments, including C, control; CF, chemical fertilizer; VC, vermicompost; SCGPF, spent coffee grounds percentage fertilizer; SCGTDF, spent coffee grounds on the top dressing fertilizer: SCGPF 5 = 5% (5 parts of SCGs and 95 parts of seed starter potting mix), SCGPF 10 = 10% (10 parts of SCGs and 90 parts of seed starter potting mix), SCGPF 25 = 25% (25 parts of SCGs and 75 parts of seed starter potting mix), and SCGPF 50 = 50% (50 parts of SCGs and 50 parts of seed starter potting mix); and SCGTDF, spent coffee grounds on the top dressing fertilizer: SCGTDF 0.5 g = 0.5 g of SCGs on the top surface layer of the soil, before each pot was irrigated with 50 mL of water; SCGTDF 1 g = 1 g of SCGs on the top surface layer of the soil, before each pot was irrigated with 50 mL of water, and SCGTDF 2.5 g = 2.5 g of SCGs on the top surface layer of the soil, before each pot was irrigated with 50 mL of water. The values are the mean ± standard error. The bars followed by the different letters are significantly different according to the Duncan’s Multiple Range Test at 0.05 level. ***: highly significant at *p* < 0.001, as revealed by the one-way analysis of variance (ANOVA).

**Figure 2:**
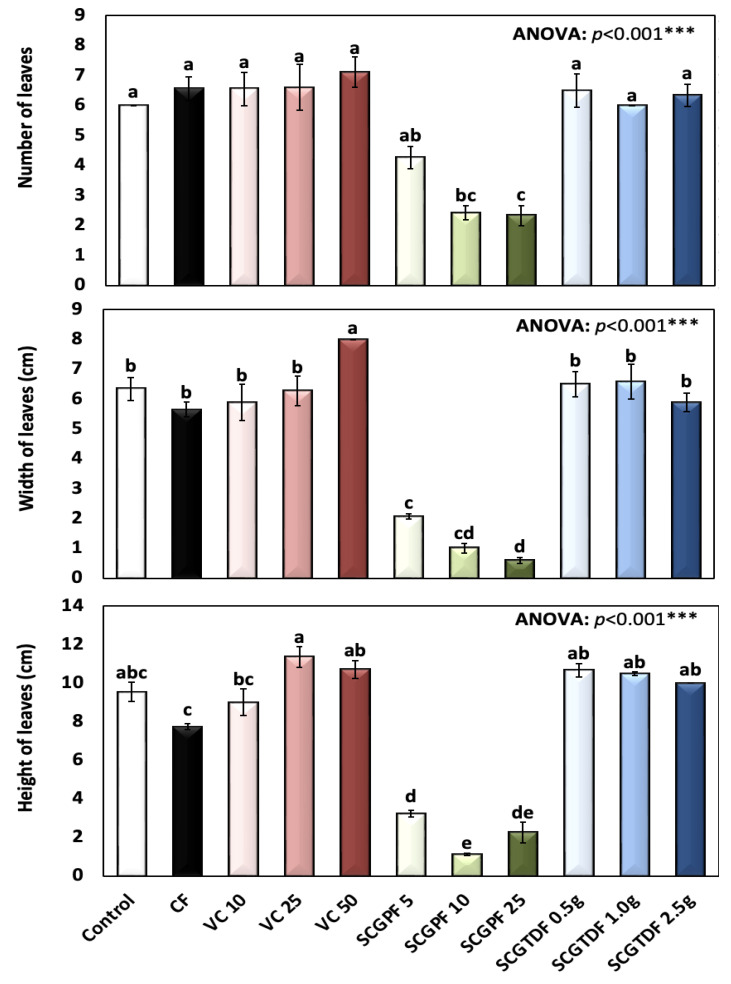
Number of leaves, width of leaves (cm), and height of leaves (cm) of *R. sativus* treated with different treatments, including C, control; CF, chemical fertilizer; VC, vermicompost; SCGPF, spent coffee grounds percentage fertilizer; and SCGTDF, spent coffee grounds on the top dressing fertilizer: SCGPF 5 = 5% (5 parts of SCGs and 95 parts of seed starter potting mix), SCGPF 10 = 10% (10 parts of SCGs and 90 parts of seed starter potting mix), SCGPF 25 = 25% (25 parts of SCGs and 75 parts of seed starter potting mix), and SCGPF 50 = 50% (50 parts of SCGs and 50 parts of seed starter potting mix); and SCGTDF 0.5 g = 0.5 g of SCGs on the top surface layer of the soil, before each pot was irrigated with 50 mL of water, SCGTDF 1 g = 1 g of SCGs on the top surface layer of the soil, before each pot was irrigated with 50 mL of water, and SCGTDF 2.5 g = 2.5 g of SCGs on the top surface layer of the soil, before each pot was irrigated with 50 mL of water. The values are the mean ± standard error. The bars followed by the different letters are significantly different according to the Duncan’s Multiple Range Test at 0.05 level. ***: highly significant at *p* < 0.001, as revealed by the one-way analysis of variance (ANOVA).

**Figure 3:**
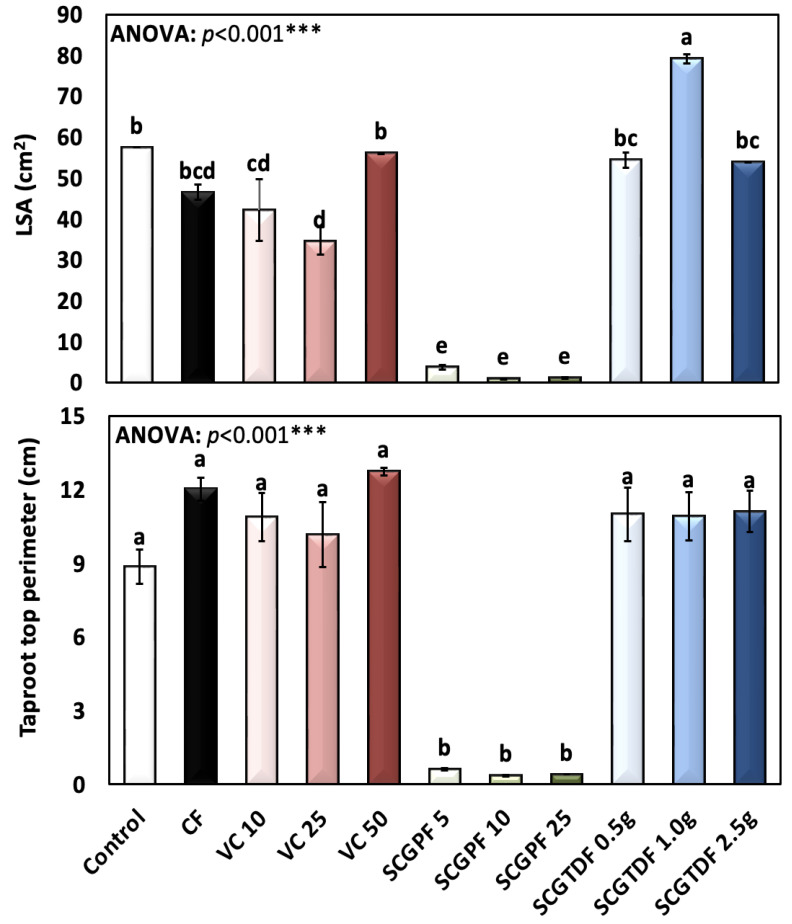
Leaf surface area (LSA) (cm^2^), and taproot top perimeter (cm) of *R. sativus* treated with different treatments, including C, control; CF, chemical fertilizer; VC, vermicompost; SCGPF, spent coffee grounds percentage fertilizer; and SCGTDF, spent coffee grounds on the top dressing fertilizer: SCGPF 5 = 5% (5 parts of SCGs and 95 parts of seed starter potting mix), SCGPF 10 = 10% (10 parts of SCGs and 90 parts of seed starter potting mix), SCGPF 25 = 25% (25 parts of SCGs and 75 parts of seed starter potting mix), and SCGPF 50 = 50% (50 parts of SCGs and 50 parts of seed starter potting mix); and SCGTDF 0.5 g = 0.5 g of SCGs on the top surface layer of the soil, before each pot was irrigated with 50 mL of water, SCGTDF 1 g = 1 g of SCGs on the top surface layer of the soil, before each pot was irrigated with 50 mL of water, and SCGTDF 2.5 g = 2.5 g of SCGs on the top surface layer of the soil, before each pot was irrigated with 50 mL of water. The values are the mean ± standard error. The bars followed by the different letters are significantly different according to the Duncan’s Multiple Range Test at 0.05 level. ***: highly significant at *p* < 0.001, as revealed by the one-way of variance (ANOVA).

**Figure 4:**
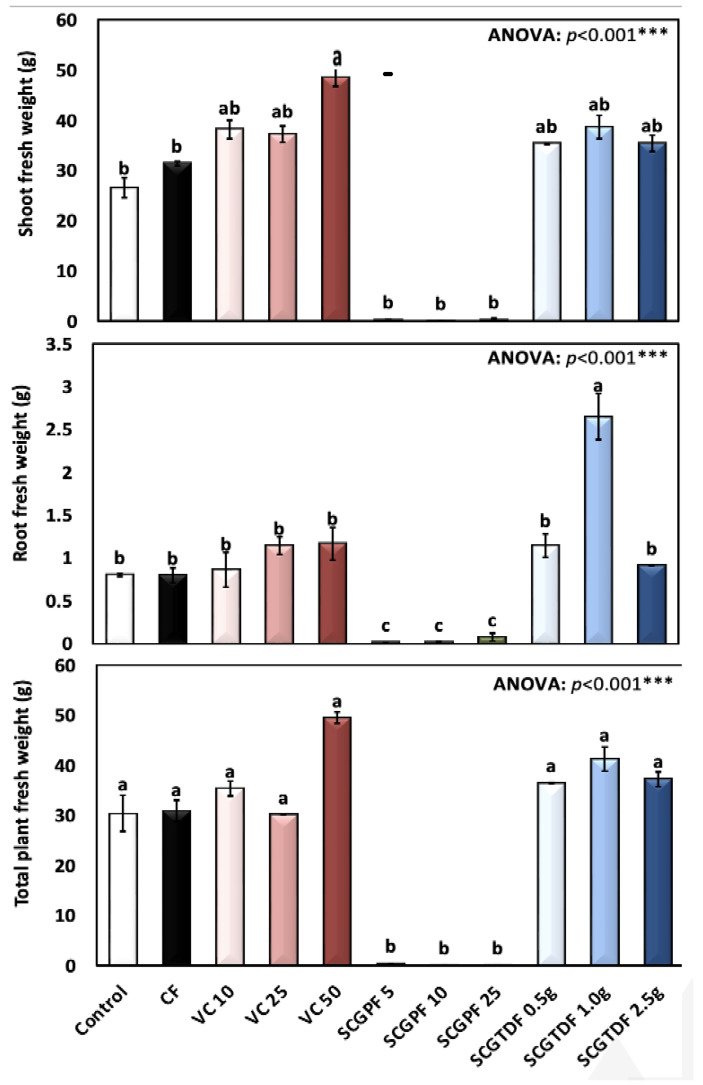
Shoot fresh weight (g), root fresh weight (g), and total plant fresh weight (g) of *R. sativus* treated with different treatments, including C, control; CF, chemical fertilizer; VC, vermicompost; SCGPF, spent coffee grounds percentage fertilizer; and SCGTDF, spent coffee grounds on the top dressing fertilizer: SCGPF 5 = 5% (5 parts of SCGs and 95 parts of seed starter potting mix), SCGPF 10 = 10% (10 parts of SCGs and 90 parts of seed starter potting mix), SCGPF 25 = 25% (25 parts of SCGs and 75 parts of seed starter potting mix), and SCGPF 50 = 50% (50 parts of SCGs and 50 parts of seed starter potting mix); and SCGTDF 0.5 g = 0.5 g of SCGs on the top surface layer of the soil, before each pot was irrigated with 50 mL of water, SCGTDF 1 g = 1 g of SCGs on the top surface layer of the soil, before each pot was irrigated with 50 mL of water, and SCGTDF 2.5 g = 2.5 g of SCGs on the top surface layer of the soil, before each pot was irrigated with 50 mL of water. The values are the mean ± standard error. The bars followed by the different letters are significantly different according to the Duncan’s Multiple Range Test at 0.05 level. ***: highly significant at *p* < 0.001, as revealed by the one-way of variance (ANOVA).

**Figure 5:**
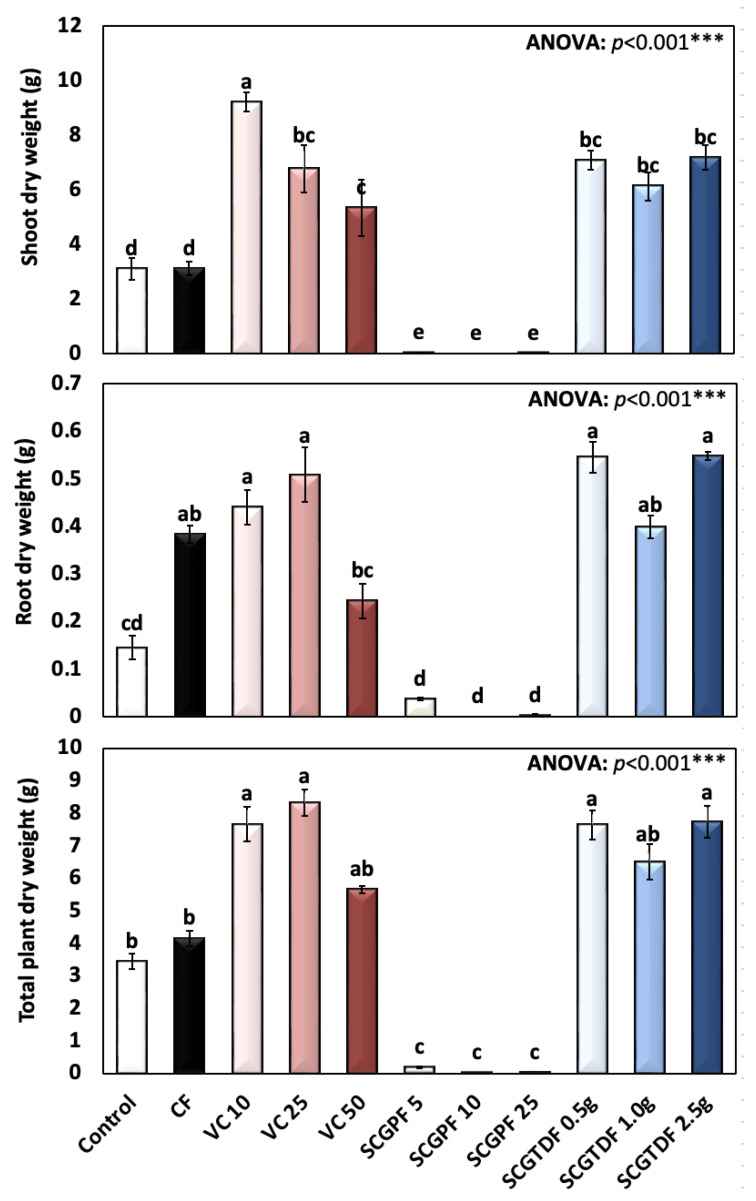
Shoot dry weight (g), root dry weight (g), and total plant dry weight (g) of *R. sativus* treated with different treatments, including C, control; CF, chemical fertilizer; VC, vermicompost; SCGPF, spent coffee grounds percentage fertilizer; and SCGTDF, spent coffee grounds on the top dressing fertilizer: SCGPF 5 = 5% (5 parts of SCGs and 95 parts of seed starter potting mix), SCGPF 10 = 10% (10 parts of SCGs and 90 parts of seed starter potting mix), SCGPF 25 = 25% (25 parts of SCGs and 75 parts of seed starter potting mix), and SCGPF 50 = 50% (50 parts of SCGs and 50 parts of seed starter potting mix); and SCGTDF 0.5 g = 0.5 g of SCGs on the top surface layer of the soil, before each pot was irrigated with 50 mL of water, SCGTDF 1 g = 1 g of SCGs on the top surface layer of the soil, before each pot was irrigated with 50 mL of water, and SCGTDF 2.5 g = 2.5 g of SCGs on the top surface layer of the soil, before each pot was irrigated with 50 mL of water. The values are the mean ± standard error. The bars followed by the different letters are significantly different according to the Duncan’s Multiple Range Test at 0.05 level. ***: highly significant at *p* < 0.001, as revealed by the one-way of variance (ANOVA).

Figure 1, Figure 2, Figure 3, Figure 4 and Figure 5 show the results of several *R. sativus* growth characteristics treated with various compost and fertilizer combinations.

This current study conducted normality tests using the Kolmogorov–Smirnov and Shapiro–Wilk tests to assess the data distribution across the various growth parameters of the red radish plants under the different fertilizer treatments. These tests were crucial for determining the appropriateness of subsequent statistical analyses, because they checked whether the data followed a normal distribution, a key assumption in many parametric tests. Accordingly, the collected data were normally distributed, and the parametric data analysis was performed.

Figure 1 presents the shoot length, root length, and total plant length of various compost and fertilizer treatments. Notably, the group treated with VC 50 showed the highest mean plant length (40.59 ± 5.7 cm), while the group with SCGPF 10 showed the lowest mean length (8.79 ± 1.52 cm) (Figure 1). The control group C (no treatment) displayed an average plant length of 35.17 ± 8.51, and the difference in the shoot length was highly significant, as revealed by the one-way ANOVA (*p* < 0.001).

Moreover, the shoot length recorded its highest value of 22.48 ± 6.2 cm in the VC 10; the lowest shoot length was 2.96 ± 0.62 cm recorded in the SCGPF 10; and the control group recorded an average shoot length of 20.62 ± 5.07 cm (Figure 1). The difference in shoot lengths was highly significant, as revealed by a one-way ANOVA (*p* < 0.001). The highest recorded root length value was 17.76 ± 4.22 cm in the VC 50, and the lowest average mean value of 6.09 ± 1.27 cm was recorded in the SCGPF 10; the difference in root lengths was highly significant, as revealed by the one-way ANOVA (*p* < 0.001). The results revealed significant variability in the shoot, root, and total plant length under different compost and fertilizer treatments (Figure 1).

The number of leaves presented in Figure 2 shows that the control group recorded an average of 6.0 ± 0.0; however, the highest number of leaves recorded was 7.11 ± 1.54 in the VC 50, and the lowest number of leaves was 2.33 ± 0.58 in the SCGPF 25, which demonstrated a highly significant difference between the treatments as revealed by the ANOVA. The highest width of the leaves was recorded in the VC 50 at 8.0 ± 0.0 cm, and the lowest number of leaves was 0.6 ± 0.17 cm in the SGPF 25. In addition, the height of the leaves showed a great variability among the treatments, with the highest mainly recorded in the VC 25 and 50 and SCGTDF 0.5, 1.0, and 2.5; however, the lowest height of the leaves was recorded in the SCGPF 5, 10, and 25 (Figure 2).

The LSA in cm^2^, and the taproot top perimeter (cm) are presented in Figure 3. The highest LSA of 79.17 ± 2.71 cm^2^ was recorded in the SCGTDF 1.0 g, and the lowest average of 0.92 ± 0.28 cm^2^ was recorded in the SCGPF 25 (Figure 3). The taproot top perimeter (cm) had a highest average value of 12.74 ± 0.46 cm and a lowest of 0.4 ± 0.0 cm. The differences in the taproot top perimeter were highly significant according to the ANOVA (*p* < 0.001) (Figure 3).

Figure 4 presents the average shoot fresh weight (g), root fresh weight (g), and total plant fresh weight (g). Generally, the lowest shoot fresh weight, root fresh weight, and total plant fresh weight were recorded in the SCGPF 5, 10, and 5. However, the highest values were observed in the VC and SCGTDF (Figure 4). The highest recorded average shoots fresh weight of 48.58 ± 5.48 g was observed in the VC 50. However, the lowest was recorded in the SCGPF 10, 25, and 5 of 0.099 ± 0.08, 0.318 ± 0.40, and 0.323 ± 0.27 g plant^−1^ fresh weight, respectively (Figure 4). In addition, the highest root fresh weight of 2.65 ± 0.66 g plant^−1^ was recorded in the SCGTDF 1.0 g and the lowest in the SCGPF 5, 10, and 25 (Figure 4).

The shoot dry weight, root dry weight, and total plant dry weight are presented in Figure 5. The highest shoot dry weight was recorded in the VC 10 (9.21 ± 1.04 g plant^−1^ DW), followed by the SCGTDF 2.0 g (7.18 ± 1.38), and the lowest of 0.0 ± 0.0 was recorded in the SCGPF 10 (Figure 5). The highest average root dry weight of 0.55 ± 0.025, and 0.55 ± 0.08 g plant^−1^ was recorded in the SCGTDF 2.0 g and 1.0 g, respectively, and the lowest was recorded in the SCGPF 10 (Figure 5). Moreover, the highest average total plant dry weights of 8.32 ± 1.31, 7.738 ± 1.49, and 7.63 ± 0.11 g plant^−1^ were recorded in the VC 25, SCGTDF 2.0 g, and SCGTDF 0.5 g, respectively (Figure 5). The differences in the shoot dry weight, root dry weight, and total plant dry weight between the fertilizers and compost were highly significant, as revealed by the ANOVA.

**Table 1:** Experimental layout under greenhouse conditions.

Experiment Replication	**Treatments**	**SCGPF**								**SCGTDF**								**VC**								**CF**								**C**							
	**Day**	**Day 0**								**Weeks 2 and 3**								**Day 0**								**Weeks 2 and 3**								**Day 0**							
	Amount	SCGPF 5%								0.5 g								VC 10								1 g of CF								C							
		1	2	3	4	5	6	7	8	1	2	3	4	5	6	7	8	1	2	3	4	5	6	7	8	1	2	3	4	5	6	7	8	1	2	3	4	5	6	7	8
		SCGPF 10%								1.0 g								VC 25																							
		1	2	3	4	5	6	7	8	1	2	3	4	5	6	7	8	1	2	3	4	5	6	7	8																
		SCGPF 25%								2.5 g								VC 50																							
		1	2	3	4	5	6	7	8	1	2	3	4	5	6	7	8	1	2	3	4	5	6	7	8																
		SCGPF 50%																																							
		1	2	3	4	5	6	7	8																																

The experiment was conducted using a split–split–plot design, where the various treatments—C, control; CF, chemical fertilizer; VC, vermicompost; SCGPF, spent coffee grounds percentage fertilizer; and SCGTDF, spent coffee grounds on the top dressing fertilizer—were randomly assigned to the subplots. Each experimental sub-plot comprised eight rows of plots, with three replicates arranged randomly. Two seeds were present in each pot. SCGPF 5 = 5% (5 parts of SCGs and 95 parts of seed starter potting mix), SCGPF 10 = 10% (10 parts of SCGs and 90 parts of seed starter potting mix), SCGPF 25 = 25% (25 parts of SCGs and 75 parts of seed starter potting mix), and SCGPF 50 = 50% (50 parts of SCGs and 50 parts of seed starter potting mix); and SCGTDF 0.5 g = 0.5 g of SCGs on the top surface layer of the soil, before each pot was irrigated with 50 mL of water, SCGTDF 1 g = 1 g of SCGs on the top surface layer of the soil, before each pot was irrigated with 50 mL of water, and SCGTDF 2.5 g = 2.5 g of SCGs on the top surface layer of the soil, before each pot was irrigated with 50 mL of water.

**Table 2:** Effect of different treatments on the soil’s pH, electrical conductivity, and macronutrients.

Parameter	pH	EC (dS/m)	Bacterial Population (CFU)	C (%)	N (%)	P (mg/kg)	K (mg/kg)
Control	6.4 ± 0.6 a	0.5 ± 0.1 c	9.0 ± 1.7 b	30.5 ± 10.1 a	1.1 ± 0.4 ab	809.2 ± 62.1 abc	2784.2 ± 698.7 abc
CF	6.7 ± 0.7 a	1.1 ± 0.1 c	5.0 ± 2.3 b	42.4 ± 15.2 a	1.1 ± 0.3 ab	549.0 ± 139.2 bc	322.0 ± 44.4 c
VC-10%	6.5 ± 0.7 a	2.0 ± 0.2 b	10.0 ± 4.0 b	39.1 ± 11.4 a	1.1 ± 0.5 ab	730.4 ± 304.1 bc	1986.9 ± 553.1 bc
VC-25%	6.7 ± 0.7 a	1.2 ± 0.1 c	38.0 ± 9.5 b	20.3 ± 3.7 a	0.7 ± 0.2 b	508.8 ± 130.5 bc	2243.4 ± 979.0 bc
VC-50%	6.8 ± 0.7 a	2.7 ± 0.3 ab	35.0 ± 14.7 b	40.8 ± 13.0 a	1.2 ± 0.2 ab	915.5 ± 205.6 abc	5157.1 ± 1956.1 a
SCGPF-5%	5.6 ± 0.6 a	2.5 ± 0.3 ab	46.0 ± 5.3 b	41.5 ± 15.1 a	1.3 ± 0.4 ab	1168.2 ± 369.6 abc	2258.0 ± 1,054.9 bc
SCGPF-10%	5.8 ± 0.6 a	3.0 ± 0.3 a	12.0 ± 4.0 b	43.7 ± 12.2 a	1.5 ± 0.4 ab	1281.2 ± 454.2 abc	2795.1 ± 798.7 abc
SCGPF-25%	5.4 ± 0.5 a	2.6 ± 0.3 ab	101.3 ± 15.5 a	43.8 ± 18.7 a	1.6 ± 0.6 a	1443.0 ± 613.6 a	3752.7 ± 1577.3 ab
SCGTDF-0.5 g	5.6 ± 0.6 a	2.5 ± 0.3 ab	4.0 ± 1.7 b	41.2 ± 9.4 a	1.1 ± 0.4 ab	420.6 ± 114.4 c	167.2 ± 75.9 c
SCGTDF-1.0 g	5.8 ± 0.6 a	3.0 ± 0.3 a	4.0 ± 0.9 b	42.8 ± 3.3 a	1.2 ± 0.5 ab	462.2 ± 203.0 c	184.3 ± 29.7 c
SCGTDF-2.5 g	5.4 ± 0.5 a	2.6 ± 0.3 ab	15.0 ± 3.1 b	41.9 ± 14.7 a	1.2 ± 0.4 ab	469.9 ± 195.2 c	200.0 ± 28.3 c
**ANOVA**	0.023 *	<0.001 **	<0.001 **	0.454 ns	0.145 ns	<0.001 **	<0.001 **

C, carbon; N, nitrogen; P, phosphorus; K, potassium; CF, chemical fertilizer; VC, vermicompost; SCGPF, spent coffee grounds percentage fertilizer; and SCGTDF, spent coffee grounds on the top-dressing fertilizer. VC-10% = 10 parts of VC product and 90 parts of seed starter potting mix; VC-25% = 25 parts of VC product and 75 parts of seed starter potting mix; and VC-50% = 50 parts of VC product and 50 parts of seed starter potting mix. SCGPF 5 = 5% (5 parts of SCGs and 95 parts of seed starter potting mix), SCGPF 10 = 10% (10 parts of SCGs and 90 parts of seed starter potting mix), SCGPF 25 = 25% (25 parts of SCGs and 75 parts of seed starter potting mix), and SCGPF 50 = 50% (50 parts of SCGs and 50 parts of seed starter potting mix); and SCGTDF 0.5 g = 0.5 g of SCGs on the top surface layer of the soil, before each pot was irrigated with 50 mL of water, SCGTDF 1 g = 1 g of SCGs on the top surface layer of the soil, before each pot was irrigated with 50 mL of water, and SCGTDF 2.5 g = 2.5 g of SCGs on the top surface layer of the soil, before each pot was irrigated with 50 mL of water—and CFU, colony forming units. The values are the means of five replicates. Within the columns, the values followed by the same letter are not significantly (*p* > 0.05) different according to the Duncan’s Multiple Range Test at 0.05 level. *, significant at *p* < 0.05; **, highly significant at *p* < 0.001; and ns, non-significant at *p* > 0.05.

**Figure 6:**
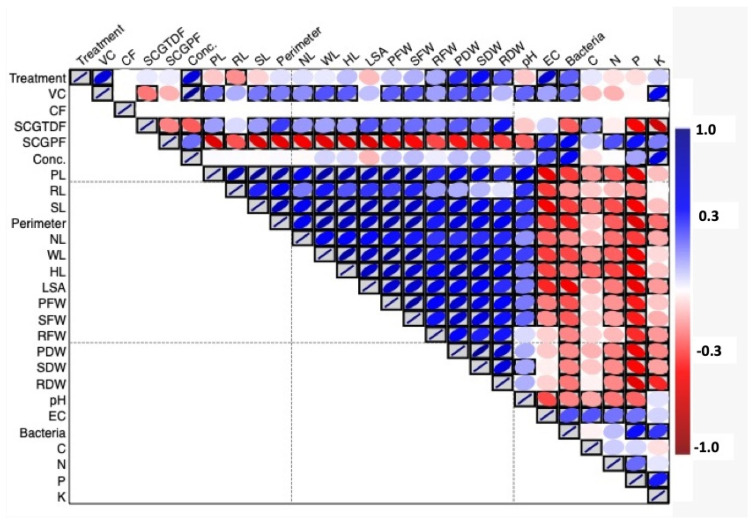
Red and blue heatmap presenting the interrelationship between the study variables including, treatment; VC, vermicompost; CF, chemical fertilizer; SCGTDF, spent coffee grounds on the top dressing fertilizer; SCGPF, spent coffee grounds percentage fertilizer; Conc., concentration; PL, plant length; RL, root length; SL, shoot length; Perimeter, taproot top perimeter; NL, number of leaves; WL, width of leaves; HL, height of leaves; LSA, leaf surface area; PFW, plant fresh weight; SFW, shoot fresh weight; RFW, root fresh weight; PDW, plant dry weight; SDW, shoot dry weight; RDW, root dry weight; EC, electrical conductivity; Bacteria, numbers of the bacterial colonies; C, carbon; N, nitrogen; P, phosphorus; and K, potassium.

The study results, as shown in Figure 6, demonstrated the connections between the different study variables using a heatmap of the Pearson’s correlation coefficients. The heatmap color scheme is as follows: red represents a negative correlation, blue represents a positive correlation, white represents no correlation, and the grey boxes indicate significant correlations. A two-tailed significance test was employed to ascertain the statistical significance of the correlation.

The VC and SCGTDF exhibited a strong positive correlation with the various growth parameters that were measured, including the shoot length, root length, plant length, taproot top perimeter, number of leaves, width of the leaves, height of the leaves, shoot fresh weight, root fresh weight, plant fresh weight, shoot dry weight, and plant dry weight. On the other hand, the SCGPF showed a clear and significant negative relationship with all the growth parameters of the shoot, root, and leaves. This is represented by the red color in the heatmap matrix. Furthermore, the SCGPF exhibited a direct relationship with the quantity of the bacterial colonies.

The heatmap also indicates the relationship between the abundance of bacterial colonies and the concentrations of macronutrients such as C, N, P, and K. The data revealed a positive correlation between the SCGPF and bacterial colonies, indicating that higher concentrations of SCGPF promote the growth of microorganisms. The SCGPF exhibited a notable rise in bacterial colony numbers, with greater concentrations (SCGPF 25% and SCGPF 50%) leading to a higher number of colonies than lower concentrations (SCGPF 5% and SCGPF 10%).

### 3.2. Effect of Different Treatments on the Soil Quality and Bacterial Population

Regarding the macronutrients, there was a clear and significant positive relationship between the SCGPF and the levels of P and K, suggesting that higher concentrations of SCGPF led to an increase in these macronutrients (Table 2). In contrast, the SCGTDF exhibited diverse associations with the macronutrient levels, demonstrating significant correlations specifically with N and C (Table 2). The findings indicate that both SCGPF and SCGTDF have varying effects on the nutrient composition of the soil and the activity of the microorganisms, emphasizing their unique functions as amendments for the soil.

The data in Table 2 summarize the impact of different treatments on the soil’s pH, EC, bacterial population, and macronutrient content. Statistically significant variations were noted in the soil’s pH (*p* = 0.023), EC (*p* < 0.001), bacterial population (*p* < 0.001), and the concentrations of P (*p* < 0.001), and K (*p* < 0.001) among the different treatments (Table 2). This illustrates the significant influence of various fertilizer types on the soil health and microbial activity.

This comprehensive analysis underscores the importance of selecting appropriate fertilizers to enhance the plant growth parameters and soil health, emphasizing the potential of SCGPF, and SCGTDF as effective soil amendments in agricultural practices.

## 4. Discussion

The organic red radish was planted and treated with different fertilizers. The VC 50% gave the highest plant growth in most of the parameters. It may be possible that the large microbial populations in the VC and the very considerable buildup of the microbial populations and activity in the soils treated with VC may have influenced the plant growth directly or indirectly [34,35]. Our results aligned with this and with a study that assessed the alterations in pH, EC, and C content in the rhizosphere of lettuce plants subjected to varying levels of VC [36].

Our results demonstrated that the application of VC led to a significant enhancement in plant height and fresh and dry weight, thereby indicating the beneficial influence of VC on plant growth. Furthermore, using VC resulted in an augmentation of the plant biomass and enhanced the soil characteristics, including the pH, organic matter content, and microbial activity [37]. The results also indicated that the application of VC led to an augmentation in both the quantity and activity of the microorganisms in the rhizosphere. This enhancement can have advantageous effects on plant development and the prevention of diseases [34,35]. Many authors have suggested that microbial activity in soils is probably responsible for improving the soil structure and indirectly influencing the root environment and plant growth, especially in 25 and 50% of VC [34,35].

This current study suggests that the type of fertilizer significantly impacts the growth of organic red radish plants. Specific treatments, like applying certain proportions of SCGs or VC, showed distinct effects on the plant growth parameters. These results have the potential to enhance sustainable farming techniques and provide insight into organic farming practices. Our results also demonstrated a significant impact of fertilizer type on the length of the red radish plants. The enormous effect size and the significant differences across the groups were found to underscore the importance of selecting appropriate fertilizer treatments to optimize plant growth [36,37]. These findings provide valuable insights for agricultural operations, particularly in optimizing the growth conditions for red radish plants and in nations that require precise fertilizer application, such as the UAE.

The fresh weight results underscore the critical impact of the fertilizer type on the fresh weight of red radish plants. The marked differences in the fresh weight across the treatments reflect the importance of choosing the appropriate fertilizers for optimal plant growth [38,39]. This present study offers valuable insights into agricultural practices, particularly for enhancing the fresh weight of red radish plants through strategic fertilizer use. The strong association between fertilizer types and fresh and dry weight outcomes suggests potential avenues for manipulating plant growth characteristics through targeted fertilization strategies [38,40,41,42].

The current results from the dry weight analysis clearly show that the fertilizer type significantly influences the dry weight of red radish plants. Much research has proved the effect of SCGs and VC on plant measurements such as weight, length, leaf development, surface area, and soil properties [7,8,37,39,41].

In the current study, SCGPF showed less effect than SCGTDF, which primarily impeded the growth of the plants. This may be attributed to two factors. Firstly, there was the stimulation of microbial growth and subsequent competition for soil nitrogen between the soil microorganisms and plant roots [39,40]. Secondly, there were the phytotoxic compounds such as polyphenols, which were present in the SCGs. Vermicomposting and pyrolysis at 400 °C have been demonstrated as effective SCG transformation methods for removing these compounds [38,41]. Nevertheless, certain studies have indicated that these compounds are accountable for the chelating characteristics of SCGs, thereby rendering their removal inadvisable. Research has also investigated the utilization of SCGs as bio-chelates, resulting in a combination of waste material and micronutrients that can be used to enhance the nutritional value of edible plants through biofortification [7,8,37,41,42].

## 5. Conclusions

In conclusion, this present study confirmed the substantial impact of the fertilizer type on the growth of organic red radish (*R. sativus*) plants, with VC at a concentration of 50% surpassing other treatments in improving the plant growth parameters. The presence of abundant microorganisms in the soils treated with VC was linked to enhanced plant height and increased fresh and dry weights. In contrast, the effectiveness of SCGPF was reduced, possibly because of competition for soil nitrogen and phytotoxic compounds in SCGs.

## 6. Future Studies

Suggestions for future studies include promoting the incorporation of VC into organic farming practices, particularly at a concentration of 25–50%, to enhance plant growth and to improve soil health.

Additional research should be conducted on the processing of SCGs to investigate their modification in order to decrease their phytotoxicity. Furthermore, their potential as a bio-chelating agent for plant biofortification should be explored.

The utilization of plant residues in their unaltered state is significantly restricted in dry environments such as the UAE, due to distinct obstacles associated with soil fertility. The UAE’s arid climate is characterized by elevated temperatures, low levels of organic matter, and inadequate soil structure, all of which substantially impact the rate of decomposition and the availability of nutrients from plant residues. These conditions cause a decelerated decomposition of organic substances, resulting in an irregular discharge of the vital nutrients necessary for ideal plant development. Moreover, the inherent high salinity and low fertility of soils in the UAE pose additional challenges when it comes to directly using plant residues without any prior treatment.

In this present study, we overcame these limitations by employing SCGs using two distinct approaches. The SCGTDF entailed applying the SCGs to the uppermost layer of the soil in varying quantities (g). The second approach utilized the SCGPF. This entailed combining precise ratios of SCGs with the seed starter potting mix, starting from the first day and using varying percentages. This research demonstrated that these modified SCGs could efficiently facilitate sustainable agricultural methods in arid areas such as the UAE by enhancing nutrient accessibility and soil composition. Integrating these insights into farming methods could significantly accelerate the advancement of sustainable agriculture in areas with limited environmental resources, such as the UAE.

## Data Availability

Further inquiries can be directed to the corresponding author.
